# Supplementary material for: Identification of a transporter complex responsible for the cytosolic entry of nitrogen-containing bisphosphonates
Source: eLife. 2018 May 10;7:e36620. doi: 10.7554/eLife.36620 (PMC6021172; doi:10.7554/eLife.36620)
Supplement: Transparent reporting form [file elife-36620-transrepform.pdf]

1st Floor
24 Hills Road
Cambridge CB2 1JP, UK

P 01223 855340
W elifesciences.org
T @elife

eLife's transparent reporting form

We encourage authors to provide detailed information *within their submission* to facilitate the interpretation and replication of experiments. Authors can upload supporting documentation to indicate the use of appropriate reporting guidelines for health-related research (see EQUATOR Network), life science research (see the BioSharing Information Resource), or the ARRIVE guidelines for reporting work involving animal research. Where applicable, authors should refer to any relevant reporting standards documents in this form.

If you have any questions, please consult our Journal Policies and/or contact us: editorial@elifesciences.org.

Sample-size estimation

- You should state whether an appropriate sample size was computed when the study was being designed
- You should state the statistical method of sample size computation and any required assumptions
- If no explicit power analysis was used, you should describe how you decided what sample (replicate) size (number) to use

Please outline where this information can be found within the submission (e.g., sections or figure legends), or explain why this information doesn't apply to your submission:

Most experiments in this study used a sample size of three. A sample size of three was chosen according to conventions in cell culture studies. Such a sample size allows for meaningful statistical analysis and visualization of out-lier data points. As we observed only small variances in out measurements, a sample size of three was able to generate highly-significant results. In cases where triplicates were not feasible (for the CRISPRi screen in Figure 1, due to an exceptionally large scale of cell culture, and for the fractionation-based uptake assay in Figure 4, due to time-sensitive steps in the protocol), duplicates were used. Again, our measurements across the replicates were sufficiently consistent to generate highly significant results. Sample size for each experiment is reported in figure legends.

Replicates

- You should report how often each experiment was performed
- You should include a definition of biological versus technical replication
- The data obtained should be provided and sufficient information should be provided to indicate the number of independent biological and/or technical replicates
- If you encountered any outliers, you should describe how these were handled
- Criteria for exclusion/inclusion of data should be clearly stated
- High-throughput sequence data should be uploaded before submission, with a private link for reviewers provided (these are available from both GEO and ArrayExpress)

Please outline where this information can be found within the submission (e.g., sections or figure legends), or explain why this information doesn't apply to your submission:

Except for the CRISPRi screens, all experiments were conducted at least twice and the results were consistent across all attempts. For each attempt replicates were included to assess the variability of results. For the alendronate CRISPRi screen, systematic comparison between measurements made in the two replicates were plotted in Figure S1a. The zoledronate screen can serve as a quasi-replicate of the alendronate screen. The number of times each experiment was attempted is included at the end of each method section.
No data were excluded.
Biological replicates refer to measurement made on several independently prepared biological samples. In tissue culture this refers to different plates of cells that were prepared separately. Technical replicates refer to multiple measurement made on a single biological sample in order to improve the precision of the measurement.

Statistical reporting

- Statistical analysis methods should be described and justified
- Raw data should be presented in figures whenever informative to do so (typically when N per group is less than 10)
- For each experiment, you should identify the statistical tests used, exact values of N, definitions of center, methods of multiple test correction, and dispersion and precision measures (e.g., mean, median, SD, SEM, confidence intervals; and, for the major substantive results, a measure of effect size (e.g., Pearson's r, Cohen's d)
- Report exact p-values wherever possible alongside the summary statistics and 95% confidence intervals. These should be reported for all key questions and not only when the p-value is less than 0.05.

Please outline where this information can be found within the submission (e.g., sections or figure legends), or explain why this information doesn't apply to your submission:

Statistical methods used in this study are described at the end of the methods section. Statistical information (including sample size, dispersion and precision measures, type of statistical test, multiple testing corrections and p-values) for each experiment is listed in figure legends.

(For large datasets, or papers with a very large number of statistical tests, you may upload a single table file with tests, Ns, etc., with reference to sections in the manuscript.)

Group allocation

- Indicate how samples were allocated into experimental groups (in the case of clinical studies, please specify allocation to treatment method); if randomization was used, please also state if restricted randomization was applied
- Indicate if masking was used during group allocation, data collection and/or data analysis

Please outline where this information can be found within the submission (e.g., sections or figure legends), or explain why this information doesn't apply to your submission:

This is not relevant to our study. We did not use animals or human participants.

Additional data files ("source data")

- We encourage you to upload relevant additional data files, such as numerical data that are represented as a graph in a figure, or as a summary table
- Where provided, these should be in the most useful format, and they can be uploaded as "Source data" files linked to a main figure or table
- Include model definition files including the full list of parameters used
- Include code used for data analysis (e.g., R, MatLab)
- Avoid stating that data files are "available upon request"

Please indicate the figures or tables for which source data files have been provided:

Source data files that contain comprehensive statistical information for the two CRISPRi screens shown in figure 1 and its figure supplement are uploaded as supplementary data files.

eLife Sciences Publications, Ltd is a limited liability non-profit non-stock corporation incorporated in the State of Delaware, USA, with company number 5030732, and is registered in the UK with company number FC030576 and branch number BR015634 at the address 1st Floor, 24 Hills Road, Cambridge CB2 1JP | August 2014
